# Resazurin-based assay for screening bacteria for radiation sensitivity

**DOI:** 10.1186/2193-1801-2-55

**Published:** 2013-02-16

**Authors:** Deborah A Hudman, Neil J Sargentini

**Affiliations:** Department of Microbiology and Immunology, AT Still University of Health Sciences, Kirksville, MO USA

**Keywords:** Resazurin, Radiation-sensitivity assay, High throughput, X-radiation, UV radiation, *Escherichia coli*

## Abstract

We report a simple and efficient colorimetric method to screen large numbers of bacterial strains for UV- and X-radiation sensitivity. We used reference radiation-sensitive and control strains of *Escherichia coli* K-12 to compare our colorimetric method to a standard clonogenic plating method. Our colorimetric method was as accurate as the standard method and was superior in terms of savings in supplies and man-hours.

## Background

Studies on radiation-sensitive mutants of bacteria, e.g., *Escherichia coli,* have been invaluable in elucidating mechanisms of DNA repair (Augusto-Pinto et al. f2003; Friedberg et al. 2006). However, it is common that one needs to screen, in time-consuming and expensive fashion, large numbers of strains to find or quantitate a desired phenotype. With this goal, we developed a resazurin-based assay using 96-well microtiter plates to reduce the very significant time and expense normally associated with traditional clonogenic (plating) assays for radiation sensitivity. We believe our new assay is sensitive, rapid, robust and economical, and it should facilitate any studies where the goal is to quickly and economically separate strains of differing radiation sensitivities, e.g., mapping or transformation studies involving hundreds of strains, or studies where large numbers of agents and concentrations would be tested for their impact on radiation survival. Conversely, our assay can be used to test for factors or mutant genotypes that might produce radiation resistance. The value of our new assay is directly proportional to number of strains or conditions that need to be tested efficiently and at low cost. Although we describe our assay using *E. coli*, this assay should be easily modifiable for use with other bacteria or higher organisms.

Resazurin is a purple, non-toxic, oxidation-reduction indicator that becomes pink when reduced to resorufin by cellular oxidoreductases (Vega-Avila and Pugsley 2011). The concentration of viable cells in a suspension containing resazurin directly determines the time-point for a visible conversion from purple to a pink color (Vega-Avila and Pugsley 2011). Resazurin reduction tests have been used for decades to demonstrate bacterial and yeast contamination of milk, and to determine chemical cytotoxicity and minimum inhibitory concentration values for antibiotics (Bigalke 1984; Drummond and Waigh 2000; McNicholl et al. 2006; Sarker et al. 2007). Resazurin has been used in a few screening studies for radiation sensitivity of mammalian cells (Gil et al. 2011; Seideman et al. 2010), however, to our knowledge, a resazurin-based assay for use in screening bacterial strains for radiation sensitivity has not been described. As in other resazurin-based studies, the readout in our assay is colorimetric and the rate of color change is directly proportional to the number of viable cells in the initial suspension. Compared to a DNA repair proficient, parental, control strain, the time-point for color conversion is extended in suspensions of cells that are more sensitive to radiation and have relatively fewer viable cells in the irradiated cell suspension. We developed this technique so that one could visually scan hundreds of microtiter wells quickly. We show that the visual results can be quantified with a microplate reader, but this is not a requirement. Visual inspection will suffice to easily identify strains that are more sensitive to radiation, i.e., their wells show more purple or less pink color than the control strain after a set time of incubation.

We report on the reliability of our colorimetric assay by testing (i) the radiation dosimetry among the 96 wells of a microtiter plate, (ii) the resazurin color change for reference radiation-sensitive and -resistant strains of *E. coli* after both UV- and X-irradiation, and (iii) the sensitivity of our colorimetric assay (an indirect measure of cell survival) in comparison with a clonogenic assay (a more traditional and direct measure of cell survival) for differentiating a set of reference *E. coli* strains based on their radiation sensitivities. We also report an estimate of the cost savings in using the colorimetric assay vs. the clonogenic assay.

## Results and discussion

**Figure 1:**
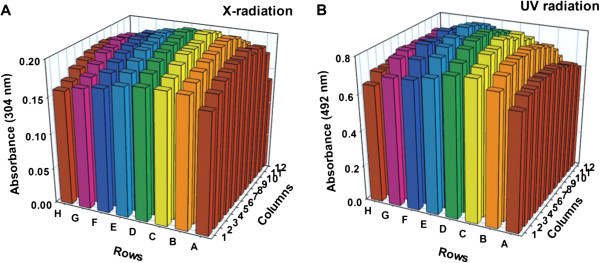
**X- and UV-radiation dose rates within the 96 wells of a microtiter plate.** (**A**) The absorbance within each well was determined at 304 nm (A_304_) in triplicate experiments using a chemical dosimeter and plotted. The rows (letters) and column (numbers) in the graph associate data with individual wells in the microtiter plate. The average dose rate for 160 kV X-rays was determined from (ΔA_304_)(280 Gy min^-1^) to be 17.96 Gy/min (sd = 0.02), and plates received a dose of 54 Gy. (**B**) A_492_ values for resazurin absorbance (indicating the cellular metabolic activity within each well) were determined from 6 experiments using a bioassay. Plates received a UV radiation dose of 50 J m^-2^. Bioassay A_492_ values were averaged and plotted. The mean value over 96 wells was 0.77 (sd = 0.02) A_492_ units.

First, we determined the well-to-well variation in X- and UV-radiation dosimetry in our 96-well microtiter plates. We used chemical dosimetry to determine the mean X-radiation dose rate over 288 wells (3 × 96), which was 17.96 ± 0.02 Gy min^-1^. The well-to-well variation of chemical dosimeter readings, which are directly proportional to X-ray dose rates, is shown in Figure 1A. Although, we measured our UV radiation dose rate at 1.42 J m^-2^ s^-1^ for the entire irradiated field, we used our resazurin-based bioassay to assess the mean effect of UV radiation on the cell suspensions in 576 wells (6 × 96), and this was 0.77 ± 0.02 A_492_ units. The well-to-well variation in UV-radiation dose rate is represented by the A_492_ values shown in Figure 1B.

**Figure 2:**
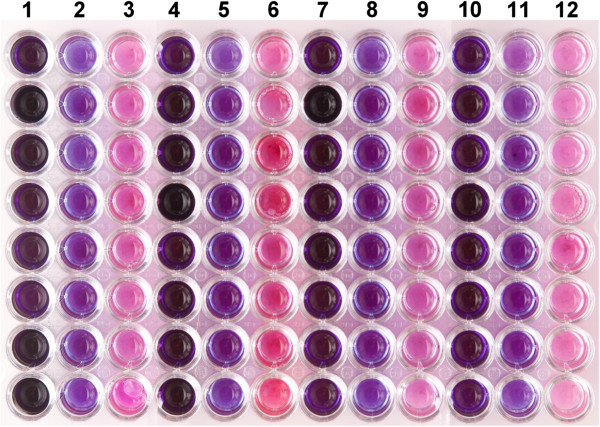
**Microtiter plate with three*E. coli*strains to demonstrate differential resazurin color change post X-irradiation.** Wells containing 50 μl of cell suspension at 6.4 × 10^7^ colony-forming units per ml were irradiated with 250 Gy, 160 μl of LB/resazurin solution were added to each well and plates were incubated at 37°C for 4.5 h. Columns 1, 4, 7, and 10 contained strain SR1252 (*polA*, higher radiation sensitivity) cells; columns 2, 5, 8, and 11 contained strain SR1553 (*recN*, intermediate radiation sensitivity); columns 3, 6, 9, and 12 contained strain SR749 (parental, control strain, lower radiation sensitivity).

Second, we visually assessed the color change after UV- or X-irradiation for 17 *E. coli* K-12 isogenic reference strains (Table 1), the DNA repair proficient, parental, control strain, SR749, and 16 others with single, radiation-sensitizing mutations at the *lexA, polA, radA, radC, recA, recB, recC, recF, recG, recJ, recN, ruvA, ruvB, umuC, uvrD,* or *ruvC* genes. During these experiments, we consistently were able to visually differentiate the 16 “radiation-sensitive” strains compared to the parental control strain based on culture color. An example of the color differential is shown in Figure 2.

**Table 1:** ***Escherichia coli*K-12 strains used in this study**^**a**^

Strains	Genotype	Source, Reference
SR749	parental, control	AB1157, E. coli Genetic Stock Center
SR1159	*recB21*	NJS Lab strain
SR1165	*umuC122*::Tn*5*	NJS Lab strain
SR1187	*radC102*	I Felszenswalb
SR1252	*polA5*	NJS Lab strain
SR1277	*uvrD254*::Tn*5*	NJS Lab strain
SR1279	*lexA101*	NJS Lab strain
SR1467	*recA srl301*::Tn*10*	NJS Lab strain
SR1553	*recN262*	NJS Lab strain
SR1643	*recC22*	NJS Lab strain
SR1663	*recJ284*::Tn*10*	NJS Lab strain
SR2384	*ruvA59*::Tn*10*	NJS Lab strain
SR2385	*ruvB60*::Tn*10*	NJS Lab strain
SR2603	Δ*ruvC64*::kan	RG Lloyd
SR2604	*recG258*::kan	RG Lloyd
SR2666	*recF332*::Tn*3*	NJS Lab strain
SR2877	*radA110*::kan	NJS Lab strain

^a^ All strains were derived from the DNA repair proficient *E. coli* K-12 AB1157 strain (SR749), and carry the following mutations: *argE3*(oc), *hisG4*(oc), *leuB6*(amIII), Δ(*gpt-proA*)*62, thr-1, thi-1, ara-14, galK2, lacY1, mtl-1, xyl-5, tsx-33, rfbD1, mgl-51, rpsL31, supE44*(amSuII), *rac,* F^-^, λ^-^. Genetic nomenclature has been described (Berlyn 1998).

**Figure 3:**
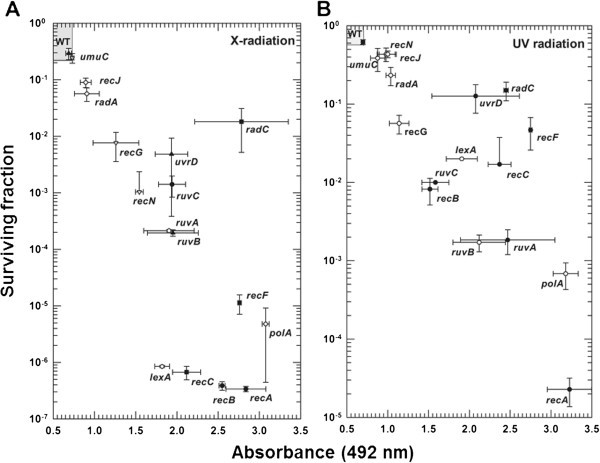
**Comparison of colorimetric and clonogenic assays for radiation-sensitive phenotype of*E. coli*reference strains.** Mutant strains listed in Table 1 were compared with the isogenic parental control strain (SR749, WT) for recovery from radiation treatment. (**A**) X-radiation, 250 Gy. (**B**) UV-radiation, 100 J m^-2^. All data points are means from triplicate experiments. Standard deviations (horizontal bars displaying variation in absorbance values and vertical bars displaying variation in surviving fraction values) are shown. The gray-shaded boxes represent the mean ± 2 sem for the WT strain. Any strain data points that fall outside of the gray-shaded boxes are significantly different (Kruskal-Wallis one way ANOVA on Ranks, P <0.05) in their absorbance and surviving fraction values from the WT strain.

The third test of the resazurin-based assay involved plotting the radiation sensitivity data (direct measure) for our set of 17 reference *E. coli* strains against resazurin/resorufin absorbance values (indirect measure) in Figures 3A and 3B. Cell surviving fractions of UV- and X-irradiated cells were determined using a clonogenic assay. Irradiated or non-irradiated cells were plated onto duplicate LB agar plates. After overnight incubation at 37°C, colonies were counted and cell-surviving fractions were calculated. Resazurin/resorufin absorbance values (A_492_) were attained from the colorimetric assay plates using a microplate reader. The results of the colorimetric and clonogenic assays are shown in Table 2, and indicate a similar ability of each assay to differentiate radiation-sensitive strains from the parental, control strain. Figures 3A and 3B confirm that irradiated strains showing lower surviving fractions (i.e., more sensitive to radiation than the parental, control strain) also showed higher A_492_ values (i.e., their irradiated cell suspensions showed less metabolic activity than the parental, control strain). We plotted the mean surviving fraction and A_492_ data (± 2 sem) for the parental, control strain (WT) to produce a gray-shaded box in the upper left-hand corner of each graph. Mean ± sd data for reference test strains that did not fall within the shaded box were considered significantly different from the parental, control strain in their A_492_ values (Kruskal-Wallis one way ANOVA on Ranks: X-radiation H = 46.891 P < 0.001, UV-radiation H = 47.370 P < 0.001). Under these constraints, only X-irradiated *umuC* cells were not different from the control strain, which was verified with a *t*-test (t = 1.912, P = 0.128). These results for *umuC* are consistent with published results for x-radiation sensitivity (Sargentini and Smith 1986). In addition, we performed a *t*-test comparing the nearest “sensitive strain”, *recJ* in this case, to the parental control strain to confirm that it was statistically different (t = 4.874, P = 0.008). For UV radiation, all test strains were significantly different from the parental, control strain and the nearest “sensitive strains” were verified with *t*-tests, *umuC* (t = 3.158, P = 0.025) and *recJ* (t = 4.775, P = 0.009). Although the data in Figures 3A and 3B suggest one could use our colorimetric assay to quantitatively differentiate *E. coli* strains on the basis of their radiation sensitivity, our focus was to develop a screening assay that would allow simple and rapid differentiation of radiation-sensitive strains from a parental, control strain.

**Table 2:** **Radiation impact on*Escherichia coli*strains assessed by clonogenic (surviving fraction) and colorimetric (resazurin) assays**^**a**^

***E. coli*** K-12 strain (DNA repair defect)	X-irradiation (250 Gy)		UV-irradiation (100 J m^-2^)	
	Surviving fraction	Resazurin A_492_	Surviving fraction	Resazurin A_492_
SR749 (parental, control)	2.9 (±0.7) e-1	0.7 (±0.04)	6.2 (±0.4) e-1	0.7 (±0.02)
SR1159 (*recB21*)	3.9 (±0.7) e-7	2.6 (± 0.04)	1.0 (±0.0) e-2	1.6 (±0.2)
SR1165 (*umuC122*::Tn*5*)	2.4 (±0.5) e-1	0.7 (±0.02)	3.9 (±1.3) e-1	0.9 (±0.09)
SR1187 (*radC102*)	2.0 (±1.0) e-2	2.8 (±0.6)	1.5 (±0.4) e-1	2.4 (±0.03)
SR1252 (*polA5*)	4.8 (±4.3) e-6	3.1 (±0.04)	6.8 (±2.5) e-4	3.2 (±0.2)
SR1277 (*uvrD254*::Tn*5*)	4.8 (±4.5) e-3	1.9 (±0.20)	1.3 (±0.5) e-1	2.1 (±0.5)
SR1279 (*lexA101*)	8.5 (±0.4) e-7	1.8 (±0.09)	2.0 (±0.0) e-2	1.9 (±0.2)
SR1467 (*recA srl301*::Tn*10*)	3.4 (±0.4) e-7	2.8 (±0.2)	2.3 (±0.9) e-5	3.2 (±0.3)
SR1553 (*recN262*)	1.0 (±1.3) e-3	1.5 (±0.05)	4.3 (±0.9) e-1	1.0 (±0.1)
SR1643 (*recC22)*	6.7 (±1.8) e-7	2.1 (±0.2)	2.0 (±2.0) e-2	2.4 (±0.1)
SR1663 (*recJ284*::Tn*10*)	9.0 (±2.0) e-2	0.9 (±0.07)	4.3 (±0.4) e-1	1.0 (±0.1)
SR2384 (*ruvA59*::Tn*10*)	2.0 (±0.3) e-4	2.0 (±0.3)	1.8 (±0.6) e-3	2.5 (±0.6)
SR2385 (*ruvB60*::Tn*10*)	2.1 (±0.09) e-4	1.9 (±0.3)	1.7 (±0.4) e-3	2.1 (±0.3)
SR2603 (Δ*ruvC64*::kan)	1.4 (±0.6) e-3	1.9 (±0.2)	8.2 (±3.1) e-3	1.5 (±0.1)
SR2604 (*recG258*::kan)	7.6 (±4.1) e-3	1.3 (±0.3)	6.0 (±2.0) e-2	1.1 (±0.2)
SR2666 (*recF332*::Tn*3*)	1.1 (±0.4) e-5	2.8 (±0.02)	5.0 (±2.0) e-2	2.8 (±0.02)
SR2877 (*radA110*::kan)	6.0 (±2.0) e-2	0.9 (±0.2)	2.3 (±0.6) e-1	1.0 (±0.06)

^a^ Data are means (± sd) from triplicate experiments. SR749 (the parental, control strain) is considered a DNA repair proficient strain (Sargentini and Smith 1986) compared to the listed 16 strains derived from it by bacteriophage transduction.

We compared our two assays for time and cost (Table 3), and found the colorimetric assay (compared to the clonogenic assay) would save about $1200 and 7 days of work per 96 *E. coli* strains tested, without considering technician pay. Therefore, our colorimetric method was superior in terms of man-hours, pipetting steps and expense for supplies when compared to the clonogenic assay.

**Table 3:** **Cost comparison of clonogenic and colorimetric assays for measuring radiation sensitivity of*Escherichia coli*strains**

Unique steps/costs^a^	Clonogenic (surviving fraction)	Colorimetric (resazurin)	Differential “cost” (clonogenic minus colorimetric)
Reusable vessels for radiation (UV, X-ray) testing of 96 strains:	Cost for 96, 50-ml centrifuge tubes (X-ray), 96 glass Petri dishes (UV) = $465	Cost for 2 microtiter plates = $6 (one-time use only)	$458
Irradiation process (strains testable/day):	20	96	Time, 5 days
Post-irradiation incubation of cell cultures	Prepare dilution blanks using buffer and reusable glass vials = $65 (100 glass vials); time, 0.5 h	None	$65; time, 0.5 h
	Prep. of agar plates $225 (for 500 Petri dishes for plating media); time, 1 day	None	$225; time, 1 day
	Plating of bacteria; time, 4 h	Adding media to wells; time, 1 h	Time, 3 h
Quantification of radiation sensitivity:	Counting colonies and calculations; time, 4 h	Read absorbance values of microtiter plate; time, 0.25 h	Time, 3.75 h
Pipetting steps (per strain tested):	6	3	3 pipetting steps per strain (i.e., more error)

^**a**^ Costs for supplies were calculated from prices listed on the Fisher Scientific website in December 2012.

## Conclusions

In summary, we have described a novel, resazurin-based colorimetric method for high-throughput screening of *E. coli* strains for radiation sensitivity. This assay is easy to follow, depends on many fewer pipetting steps, is highly economical in terms of man-hours and supplies, and provides results that compare well with standard, more expensive and time-consuming clonogenic assays.

## Methods

X-radiation dose rates were determined for a Polaris Model XR160 cabinet irradiator (Kimtron) using Fricke’s dosimetry solution (0.8 N H_2_SO_4_, 1 mM FeSO_4_∙ 7H_2_O, 1 mM NaCl) (Fricke and Hart 1966). Fricke’s solution was placed in a 96-well microtiter plate (Fisherbrand round bottom, Fisher Scientific) at 200 μl/well and irradiated for 3 min at the center of a metal platform 14 cm below a 3000 W, Varian NDI-161 tube running at 160 kV and 15 mA. The dose rate in each well was determined by measuring the absorbance at 304 nm (A_304_) using a biophotometer (Eppendorf). The mean dose rate over the 96 wells of triplicate plates was calculated from (ΔA_304_)(280 Gy min^-1^) for 160 kV X-rays (Shalek and Smith 1969) to be 17.96 Gy/min (sd = 0.02).

UV radiation was supplied by an 8-W germicidal lamp (GE, G8T5) emitting primarily at 254 nm. The UV radiation dose rate was 1.42 J m^-2^ s^-1^ at the base of a microtiter plate (47 cm below the lamp) using a germicidal photometer (Model IL1700, International Light, Inc.). However, we used a bioassay to test for uniformity of dose rate across the 96 wells of a microtiter plate. For this purpose, *E. coli* strain SR749 was grown overnight in 5 ml Luria-Bertani (LB) broth (Miller 1972) supplemented with 1% glucose for 15–17 h (in a tube roller for aeration) to a stationary-phase cell concentration of ~1 × 10^9^ colony-forming units (CFU) per ml. Cultures were diluted ~15-fold to an optical density at 600 nm (OD_600_) of 0.03 (NanoDrop 2000c; Fisher; corresponding to 6.4 × 10^7^ CFU/ml) with 67 M NaK phosphate buffer (PB). A 50-μl cell volume was placed into each well of six 96-well microtiter plates and these were UV-irradiated with a dose of 50 J m^-2^. After irradiation, 150 μl of LB and 10 μl of 0.675% resazurin (Difco) solution were added to each well and plates were incubated at 37°C for 4.5 h. To compare the UV radiation doses received in each of the 96 wells of the microtiter plates, we determined the development of pink color (related to cell viability) by measuring A_492_ values (LabSystems MultiSkan MCC/340).

To simultaneously measure radiation sensitivity of *E. coli* strains by resazurin and clonogenic assays, cells were prepared as above, but 300-μl volumes of cell suspensions (~6.4 × 10^7^ CFU/ml) were placed in wells in two separate 96-well microtiter plates. One plate was X-irradiated (250 Gy), the other was UV irradiated (100 J m^-2^). After irradiation, cells were re-pipetted (3x) and 250 μl of cells from each well were removed and saved for the clonogenic assay (along with a sample of non-irradiated cells). The remaining 50-μl volume of cells was mixed with LB/resazurin solution and incubated as above. The incubation time (4.5 h) was optimized in pilot experiments to consistently be able to visually differentiate cultures of control radiation-resistant strains (bright pink color) from cultures of radiation-sensitive strains (purple color). Once incubation was complete, A_492_ values were determined as above. Cell surviving fractions of UV- and X-irradiated cells were determined using our clonogenic assay by plating the cells saved from the microtiter plates used in the colorimetric assay. Irradiated or non-irradiated cells were spread onto duplicate LB agar plates, either directly or after dilution in PB. After overnight incubation at 37°C, colonies were counted to determine the colony forming units per ml (CFU/ml) values for the non-irradiated and irradiated cell suspensions. The CFU/ml value for a cell suspension was determined by multiplying the mean number of colonies per plate by the dilution factor. The cell surviving fraction was determined as the ratio of the CFU/ml value after each radiation dose divided by the CFU/ml value for non-irradiated (control) cells. Experiments were completed in triplicate to determine the mean surviving fraction ± sd for each radiation dose.
